# The Noradrenaline Metabolite MHPG Is a Candidate Biomarker from the Manic to the Remission State in Bipolar Disorder I: A Clinical Naturalistic Study

**DOI:** 10.1371/journal.pone.0100634

**Published:** 2014-06-27

**Authors:** Masatake Kurita, Satoshi Nishino, Yukio Numata, Yoshiro Okubo, Tadahiro Sato

**Affiliations:** 1 Sato Hospital, Koutokukai, 948-1 Kunugizuka, Nanyo, Yamagata, Japan; 2 Department of Cellular Signaling, Graduate School of Pharmaceutical Sciences, Tohoku University, 6-3 Aoba, Aramaki, Aoba-ku, Sendai, Japan; 3 Department of Psychiatry and Behavioral Science, Graduate School of Medicine, Nippon Medical School, Bunkyo-ku, Tokyo, Japan; University of Illinois at Chicago, United States of America

## Abstract

Remission is the primary goal of treatment for bipolar disorder I (BDI). Metabolites of noradrenaline and dopamine, 3-methoxy-4-hydroxyphenylglycol (MHPG) and homovanillic acid (HVA), respectively, are reduced by treatment with antipsychotics, but whether these phenomena are caused by antipsychotics or by the pathophysiology of BDI is not known. Interactions between brain-derived neurotrophic factor (BDNF) and mood disorders have also been suggested. We conducted a multifaceted study in BDI patients to ascertain if biological markers are associated with the manic state. Patients with Young Mania Rating Scale (YMRS) scores >20 participated in the study. Final analyses involved 24 BDI patients (13 men and 11 women). We used YMRS scores to identify mania stages in individual BDI patients (i.e., manic syndrome, response and remission stages). Statistical analyses were done using one-way repeated-measures analyses of variance (rep-ANOVA) throughout manic syndrome, response and remission stages. Plasma concentrations of MHPG and HVA were analyzed by high-performance liquid chromatography with electrochemical detection. Plasma levels of BDNF were measured by sandwich enzyme-linked immunosorbent assay. BDI patients had significantly reduced plasma levels of MHPG throughout manic syndrome, response and remission stages (rep-ANOVA, *p* = 0.002). Without a case of response state, there was a significant positive correlation between YMRS scores and plasma levels of MHPG (ρ = 0.33, *p* = 0.033, n = 48). Plasma levels of HVA and BDNF were not significantly altered throughout manic syndrome, response and remission stages. These data suggest that the peripheral level of MHPG (which is associated with noradrenaline levels in the brain) could be used as a biomarker for the manic state in BDI. The MHPG level is likely to reflect the clinical characteristics of the manic syndrome in BDI, and noradrenaline may reflect the pathophysiology from manic to remission states.

## Introduction

Bipolar disorder I (BDI) is an episodic illness characterized by recurrent manic, mixed, and depressive episodes with an estimated global lifetime prevalence of 1–5% [1]. BDI is associated with a high prevalence of mortality and morbidity [2], functional impairment [3], and suicide [4]. Mania is a core aspect of the presentation of BDI, but the pathophysiology of manic syndrome is poorly understood. Recent evidence suggests that atypical antipsychotics are effective for the treatment of manic syndrome [5], [6]. Moreover, it is known that levels of the metabolites of noradrenaline and dopamine, 3-methoxy-4-hydroxyphenylglycol (MHPG) and homovanillic acid (HVA), respectively, are higher in bipolar manic patients more than in normal control [7], [8], are reduced by treatment with antipsychotic agents [8]. However, several reports have suggested that antipsychotics can induce depression or extrapyramidal symptoms (EPS) in bipolar manic patients [9], [10], [11]. Thus, whether the decreased levels of MHPG and HVA are caused by the carryover effect of antipsychotics or by the pathophysiology of BDI is not known.

Furthermore, a meta-analysis showed that brain-derived neurotrophic factor (BDNF) levels were decreased in bipolar mania and bipolar depression when compared with both control groups [12]. It is also not clear whether the decreased levels of BDNF are caused by the carryover effect of antipsychotics or by the pathophysiology of BDI. Thus, the association of BDI with peripheral biomarkers in the pathophysiology of BDI is not clear. Currently, there is no biomarker that could serve as an objective index for evaluating the severity of BDI or whether a particular treatment will be effective for BDI.

The main purpose of the present study was to ascertain if plasma levels of catecholamine metabolites and BDNF are correlated with the severity of manic symptoms in BDI, and to understand the pathophysiology of BDI. We focused on the correlation of biomarkers and symptoms, not the effects of medication upon biomarkers. Our report is the first naturalistic, retrospective study examining levels of MHPG (a noradrenaline metabolite associated with noradrenaline levels in the brain), HVA (a dopamine metabolite associated with dopamine levels in the brain) and BDNF in manic states of BDI.

## Methods

### Subjects

**Figure 1:**
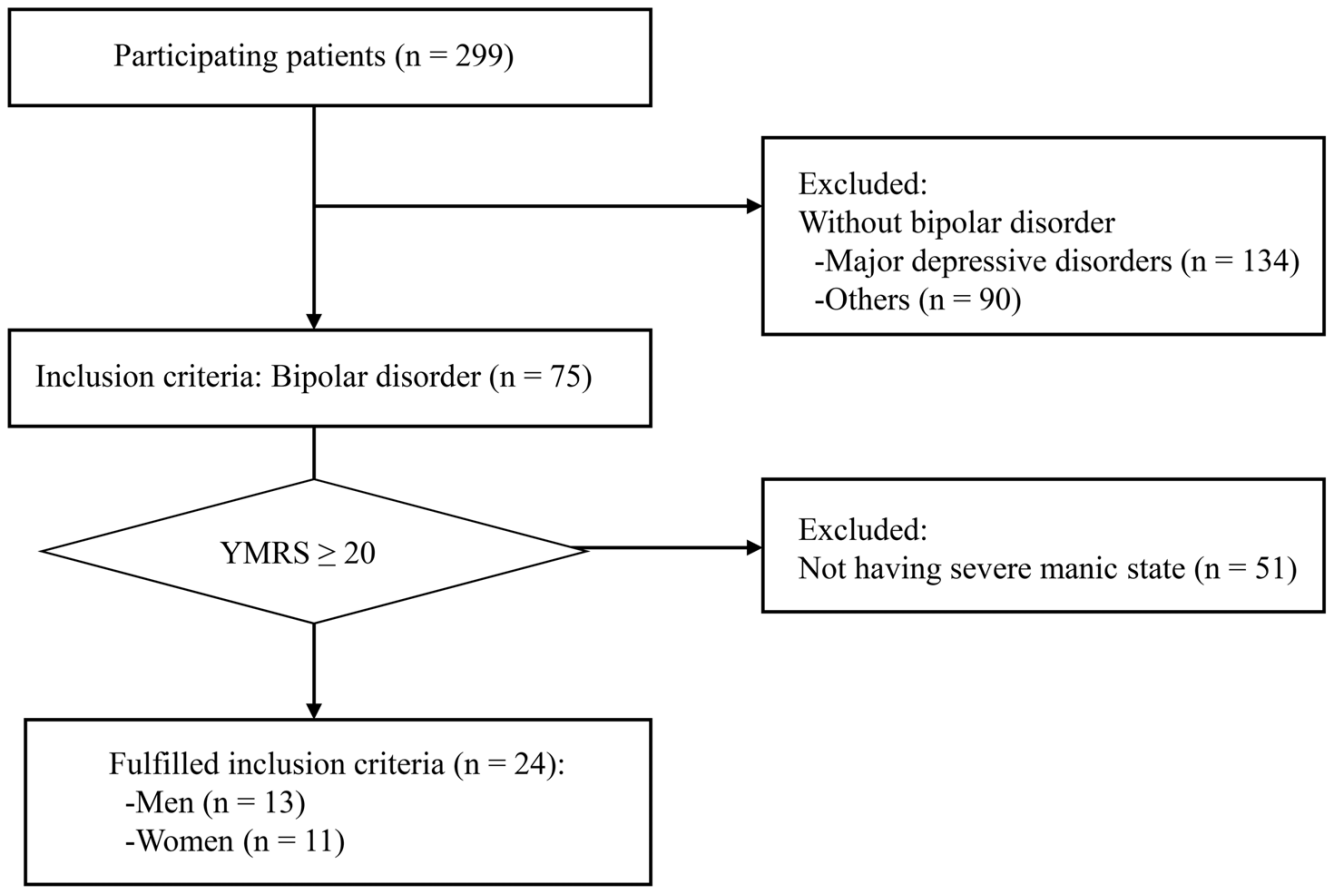
Flowchart of patient numbers through the study.

A detailed flowchart of patient selection is shown in Fig. 1. Subjects were recruited from BPI patients admitted to the inpatient and outpatient clinics of Sato Hospital (Koutokukai, Japan) between June 2006 and August 2010. All patients were suffering from a manic episode according to the diagnosis of BDI stated in the *Diagnostic and Statistical Manual for Mental Disorders* (fourth edition, text version, DSM-IV-TR, American Psychiatric Association, 2000). Subjects with any other diagnosed mental or severe physical illness were excluded from the study. Patients were also excluded if they exhibited a borderline personality disorder, antisocial personality disorder, or if they had a history of substance dependence.

After the procedures had been explained fully to them, participants provided written informed consent. Study protocols were approved by the Ethics Committee of Sato Hospital and the Ethics Committee of the Graduate School of Pharmaceutical Sciences of Tohoku University (Sendai, Japan). Standard procedures for clinical trials involving vulnerable participants in Japan were followed. This study was undertaken according to the ethical standards set in the Declaration of Helsinki. Patients who declined to participate or who otherwise did not take part remained eligible for treatment, and were not disadvantaged in any way by not participating in the study.

As a criterion for patient inclusion, we chose a Young Mania Rating Scale (YMRS) score of ≥20 points as the manic syndrome stage [13]. In addition, patients were considered to be in remission if they scored ≤8 on the Montgomery–Åsberg Depression Rating Scale (MADRS) [14] and YMRS [15], [16]. The response stage was defined as a reduction of <50% in YMRS score from the manic syndrome stage. The final analyses comprised 24 BDI patients (13 men and 11 women; average age, 54.5±16.0 (range, 24–79) years).

None of the subjects were taking hormone therapies (including oral contraceptives). Dosage and administration of antihypertensive medications were unchanged during this study. The following antihypertensive medications were administered during this study: amlodipine (5 mg/day; n = 2), lisinopril (20 mg/day; n = 1) and nifedipine (20 mg/day; n = 1). One patient was administered acetaminophen (450 mg/day; n = 1) temporarily during the responder state. Most patients (22/24) had been prescribed mood stabilizers and/or antipsychotic drugs by psychiatrists. Psychiatrists treated patients with brief sessions of psychotherapy. The following mood stabilizers and antipsychotic drugs were administered in the manic syndrome stage: lithium (200–800 mg/day; n = 8), valproic acid (400–1600 mg/day; n = 16), lamotrigine (100 mg/day; n = 1), carbamazepine (400 mg/day; n = 1), risperidone (1.5–4 mg/day; n = 4), quetiapine (50–200 mg/day; n = 2), chlorpromazine (12.5–150 mg/day; n = 3), propericiazine (10–30 mg/day; n = 2), levomepromazine (5–50 mg/day; n = 6), zotepine (150 mg/day; n = 1) and aripiprazole (18 mg/day; n = 1). The mean dose at the syndrome stage for chlorpromazine equivalence [17] was 116±145 mg/day. Eight patients were not taking antipsychotics at the syndrome stage. The following mood stabilizers and antipsychotic drugs were administered in the response stage: lithium (300–800 mg/day; n = 8), valproic acid (200–2000 mg/day; n = 17), lamotrigine (100 mg/day; n = 1), carbamazepine (400 mg/day; n = 2), risperidone (1.5–16 mg/day; n = 3), quetiapine (50–600 mg/day; n = 2), chlorpromazine (37.5 mg/day; n = 1), propericiazine (15 mg/day; n = 1), levomepromazine (5–150 mg/day; n = 6), zotepine (50–400 mg/day; n = 2), aripiprazole (12 mg/day; n = 1), blonanserin (4 mg/day; n = 1) and sultopride (300 mg/day; n = 1). The mean dose at the response stage for chlorpromazine equivalence was 187±473 mg/day. Eleven patients were not taking antipsychotics at the response stage. The following mood stabilizers and antipsychotic drugs were administered in the remission stage: lithium (300–800 mg/day; n = 9), valproic acid (200–2000 mg/day; n = 18), lamotrigine (100 mg/day; n = 1), carbamazepine (400 mg/day; n = 1), risperidone (0.5–16 mg/day; n = 3), quetiapine (50 mg/day; n = 1), propericiazine (10 mg/day; n = 1), levomepromazine (5–80 mg/day; n = 5), zotepine (50–400 mg/day; n = 2), aripiprazole (12 mg/day; n = 1) and sultopride (100 mg/day; n = 1). The mean dose at the remission stage for chlorpromazine equivalence was 134±448 mg/day. Thirteen patients were not taking antipsychotics at the remission stage (Table 1).

**Table 1:** Medications taken by study patients.

	Syndrome	(mg)	Response	(mg)	Remission	(mg)
Case 1	-		-		-	
Case 2	lithium	200	valproic acid	200	valproic acid	200
	valproic acid	600				
	risperidone	4				
Case 3	lithium	800	lithium	800	lithium	800
	valproic acid	800	valproic acid	1200	valproic acid	1200
	levomepromazine	50	levomepromazine	50	levomepromazine	50
Case 4	-		-		-	
Case 5	valproic acid	1200	lithium	600	lithium	600
	chlorpromazine	150	valproic acid	1200	valproic acid	1200
			aripiprazole	12	aripiprazole	12
Case 6	lithium	800	lithium	800	lithium	800
	chlorpromazine	37.5	chlorpromazine	37.5	chlorpromazine	10
	levomepromazine	10	levomepromazine	10		
Case 7	lithium	300	carbamazepine	400	valproic acid	400
	aripiprazole	18				
Case 8	lithium	500	lithium	600	lithium	600
	valproic acid	600	valproic acid	800	valproic acid	800
	risperidone	1.5	risperidone	1.5	risperidone	2
Case 9	valproic acid	1000	valproic acid	1600	valproic acid	1600
Case 10	lithium	600	lithium	400	lithium	400
	valproic acid	600	valproic acid	600	valproic acid	600
	propericiazine	30	propericiazine	15	propericiazine	10
	levomepromazine	50	sultopride	300	sultopride	100
			levomepromazine	150	levomepromazine	75
Case 11	lithium	400	lithium	400	lithium	400
	valproic acid	800	valproic acid	400	valproic acid	400
	propericiazine	10	levomepromazine	10		
	levomepromazine	25				
Case 12	valproic acid	1600	valproic acid	2000	valproic acid	2000
	quetiapine	200	quetiapine	600		
Case 13	lamotrigine	100	lamotrigine	100	lamotrigine	100
	quetiapine	50	quetiapine	50	quetiapine	50
Case 14	valproic acid	1200	valproic acid	800	valproic acid	800
Case 15	lithium	800	lithium	400	lithium	400
	valproic acid	1000	valproic acid	1000	valproic acid	1000
	chlorpromazine	12.5				
Case 16	-		valproic acid	1000	valproic acid	1200
Case 17	carbamazepine	400	carbamazepine	400	carbamazepine	400
	levomepromazine	35	levomepromazine	80	lithium	600
					levomepromazine	80
Case 18	valproic acid	1400	valproic acid	1000	valproic acid	1000
Case 19	valproic acid	1200	valproic acid	1500	valproic acid	1400
Case 20	valproic acid	1000	valproic acid	1200	valproic acid	1200
	levomepromazine	5	blonanserin	4	levomepromazine	5
			levomepromazine	5		
Case 21	risperidone	2	risperidone	1	risperidone	0.5
Case 22	valproic acid	900	valproic acid	900	valproic acid	900
Case 23	valproic acid	1100	lithium	300	lithium	300
	zotepine	150	valproic acid	1200	valproic acid	1200
			zotepine	50	zotepine	50
Case 24	valproic acid	400	valproic acid	1600	valproic acid	1600
	risperidone	4	risperidone	16	risperidone	16
			zotepine	400	zotepine	400

### Assessment

The clinical status of patients was evaluated using the YMRS [15] and MADRS [14]. Symptom severity was assessed every two weeks by independent experienced raters using the YMRS and MADRS. Raters were blinded to the treatment the participants had been receiving and were not associated with treatment administration.

### Sample Collection

Blood sampling and mood assessments were carried out at the same time of day every two weeks. Blood was drawn from each subject by venipuncture into a blood-collection tube containing the anticoagulant ethylenediamine tetra-acetic acid between 10:00 and 17:00. Tubes were cooled immediately to 4°C and then centrifuged at 2000× *g* for 20 min. Plasma was kept at −80°C until assay.

### Laboratory Assays

Plasma levels of MHPG and HVA were analyzed by high-performance liquid chromatography with electrochemical detection [18], [19] using internal-standard (5-hydroxyindolecarboxylic acid) and standard-addition methods. Plasma levels of BDNF were analyzed by sandwich enzyme-linked immunosorbent assay as described previously [20].

### Data Analyses

Statistical analyses of MADRS and YMRS scores and plasma levels of MHPG, HVA and BDNF were undertaken using one-way repeated-measures analyses of variance (rep-ANOVA) for the three stages of symptoms. *Post hoc* tests were carried out on rep-ANOVA results using the Bonferroni correction for multiple comparisons. Data are the means ± standard deviation (mean ± SD). Correlation analyses were done using Spearman's correlation (ρ). *p*<0.05 was considered significant. Testing for normal distribution of data was undertaken using Mauchly's sphericity test. Analyses were carried out using SPSS v16.0 (SPSS, Chicago, IL, USA).

### Trial Registration

This study is registered in the UMIN Clinical Trials Registry (UMIN-CTR): Analysis of genome and blood components for elucidation and treatment of mood disorders (ctr.cgi&quest;function&hairsp;&equals;&hairsp;brows&amp;action&hairsp;&equals;&hairsp;brows&amp;recptno&hairsp;&equals;&hairsp;R000007415&amp;type&hairsp;&equals;&hairsp;summary&amp;language&hairsp;&equals;&hairsp;E; UMIN000006264).

## Results

### Assessment

We used the YMRS score to separate stages (i.e., manic syndrome, response, remission). The manic syndrome stage was defined as a YMRS of ≥20 points [13]. The response stage was defined as a reduction in the YMRS score of <50% from the manic syndrome stage. The remission stage was defined as a YMRS score ≤8 [15], [16]. Mean YMRS scores for manic syndrome, response, and remission stages were 32.5±8.3, 6.4±5.9, and 1.9±2.7, respectively. Consequently, patients in the remission group had significantly reduced YMRS scores during treatment (repeated-measures ANOVA; F_2,46_ = 224.823, *p*<0.001). There were significant differences in YMRS scores among stages (manic syndrome *vs* response (*p*<0.001), manic syndrome *vs* remission (*p*<0.001), and response *vs* remission (*p* = 0.007); Bonferroni's multiple comparison). Patients exhibited no significant changes in MADRS score among manic syndrome, response and remission stages (0.7±1.5, 1.5±2.4, and 1.7±2.3, respectively) (repeated-measures ANOVA; F_2,46_ = 2.310, *p* = 0.111). The period from the manic syndrome stage to the response stage was 3.4±2.4 weeks. The period from the manic syndrome stage to the remission stage was 5.8±3.2 weeks (Table 2).

**Table 2:** Mean changes in outcome measures.

	Syndrome	Response	Remission	*p*	
Period from the syndrome (weeks)		3.4 (2.4)	5.8 (3.2)		
Assessments					
**YMRS** (points)	32.5 (8.3)	6.4 (5.9)	1.9 (2.7)	<0.001	***
**MADRS** (points)	0.7 (1.5)	1.5 (2.4)	1.7 (2.3)	0.111	
Biological markers					
**MHPG** (ng/mL)	10.14 (2.85)	9.70 (2.93)	8.33 (2.20)	0.002	**
**HVA** (ng/mL)	11.54 (4.36)	11.72 (7.65)	10.02 (4.44)	0.273	
**BDNF** (pg/mL)	5493 (4905)	6611 (4822)	6619 (4709)	0.293	

Values are the mean (SD).

Statistical analyses were carried out using one-way repeated measures analysis of variance.

Statistically significant (**p<0.01, ***p<0.001).

YMRS, Young Mania Rating Scale; MADRS, Montgomery–Åsberg Depression Rating Scale; MHPG, 3-methoxy-4-hydroxyphenylglycol; HVA, homovanillic acid;

BDNF, brain-derived neurotrophic factor.

### Biological Markers

**Figure 2:**
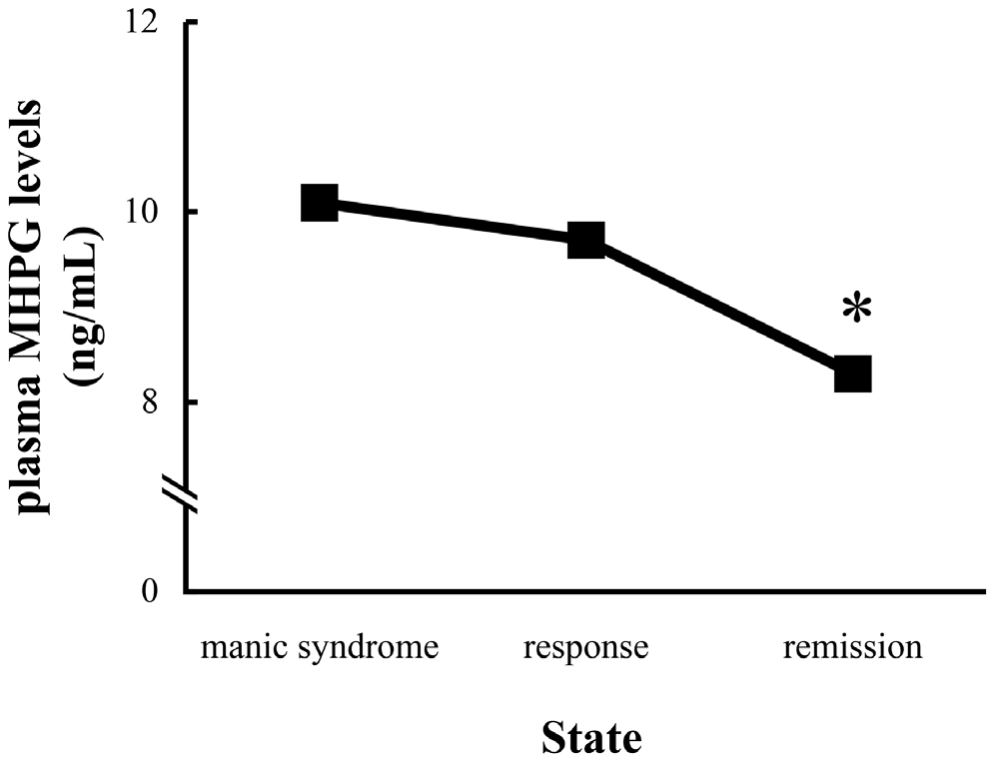
Changes in plasma levels of MHPG in the manic state for BDI patients. Each point represents the mean value. The significance of differences was calculated using repeated-measures ANOVA with *post hoc* Bonferroni testing (*p<0.05). Patients had significantly reduced plasma levels of MHPG throughout the manic syndrome, response and remission stages (repeated-measures ANOVA; F_2,46_ = 7.370, *p* = 0.002).

Plasma levels of MHPG in manic syndrome, response and remission stages were 10.1±2.9, 9.7±2.9, and 8.3±2.2 ng/mL, respectively. Patients had significantly reduced plasma levels of MHPG throughout the manic syndrome, response and remission stages (repeated-measures ANOVA; F_2,46_ = 7.370, *p* = 0.002) (Fig. 2). There were significant differences in plasma levels of MHPG among stages (manic syndrome *vs* response (*p* = 0.871), manic syndrome *vs* remission (*p* = 0.006), and response *vs* remission (*p* = 0.054); Bonferroni's multiple comparison).

Treatment of BDI led to an expected decrease in YMRS scores, and this was accompanied by a significant decrease in plasma levels of MHPG. A trend for positive correlation was found between YMRS scores and plasma levels of MHPG throughout the manic syndrome, response and remission stages (ρ = 0.214, *p* = 0.071, n = 72). In the case of taking into account only the manic syndrome and remission stages, there was a significant positive correlation between YMRS scores and plasma levels of MHPG (ρ = 0.33, *p* = 0.033, n = 48). In contrast, patients exhibited no significant changes in plasma levels of HVA or BDNF throughout the manic syndrome, response and remission stages (repeated-measures ANOVA; F_2,46_ = 1.334, *p* = 0.273; F_2,46_ = 1.261, *p* = 0.293). No significant correlation was found between plasma levels of HVA or BDNF and the manic state (Table 2).

## Discussion

In the present study, we focused on the pathophysiology of BDI. An advantage of our naturalistic study was that we could compare the multifaceted biomarkers between manic states. Antipsychotics [21] and mood stabilizers [22] have good efficacy in controlling manic syndrome in BDI patients.

Several reports have suggested that the BDNF system might change individual susceptibility to BDI [12], [23]. Also, some studies have shown levels of MHPG and HVA to be decreased after antipsychotic treatments in BDI patients [8] and psychotic patients [24]. Thus, the association between BDI and peripheral biomarkers is not consistent with respect to the pathophysiology of BDI.

For the first time in the literature, patients in the manic state of BDI underwent evaluation of levels of MHPG, HVA and BDNF in a naturalistic study. Interestingly, BDI patients had significantly reduced plasma levels of MHPG throughout manic syndrome, response and remission stages. Conversely, no significant changes were observed between plasma levels of HVA or BDNF (Table 2). In the case of taking into account only the manic syndrome and remission stages, we found a significant positive correlation between YMRS scores and plasma levels of MHPG. However, only a trend for positive correlation was found between YMRS scores and plasma levels of MHPG within the three states. BDI patients had significantly reduced plasma levels of MHPG throughout the three states, but the response state may be dependent upon the individual. These results suggest that the plasma level of MHPG acted as a peripheral biomarker, consistently indicating the synchronized progression of symptoms from the manic state to the remission state in BDI. MHPG is the major metabolite of noradrenaline in the brain [25]. In the present study, only the plasma level of MHPG exhibited significant changes that correlated with the YMRS score among stages. The results of the present study suggest that changes in the noradrenergic system are more likely than changes in the dopaminergic and BDNF systems to improve manic syndrome in BDI.

Antipsychotics can induce depression or EPS in bipolar manic patients [9], [10], [11]. Hence, antipsychotics are a temporary treatment in severe manic cases, and clinicians know that there is an increased risk of depression or EPS if high doses of antipsychotics are continued to be prescribed. In the present study, the mean dose at remission for chlorpromazine equivalence [17] was 134±448 mg/day. In studies involving decreased levels of MHPG and HVA, the mean doses for chlorpromazine equivalence [17] were 380±110 and 300–600 (for a 60-kg body weight) mg/day, respectively [8], [24]. Further studies are needed to ascertain if decreased levels of MHPG and HVA are due to the carryover effects of antipsychotics. With respect to chlorpromazine equivalence, this study suggests that the effects of antipsychotics are slight. In addition, of the 24 patients in the present study, the number of subjects not taking antipsychotics increased from 8 patients in the syndrome stage to 13 patients in the remission stage. In summary, the result of decreased plasma levels of MHPG may reflect the pathophysiology from manic to remission states.

This study had limitations. One was the lack of a control group with placebo treatments. However, even though most treatments involved mood stabilizers in the remission state, plasma levels of MHPG continued to decline. Blood was drawn from individual patients in the same time zone as much as possible, but could not be withdrawn from all patients in the same time zone. Plasma levels of MHPG do not change a great deal within the active phase [26]. On the other hand, phase advance in diurnal MHPG rhythm repoted in depression [27]. Conversely, diurnal variation in plasma levels of BDNF may be associated with differences between the sexes [28], [29]. The half-life in the human brain is 88.4 h for olanzapine and 45.5 h for risperidone [30], so the carryover effects of antipsychotics may not have occurred in the present study. More large-scale design follow-up studies are needed to understand the pathologic state for the treatment of BDI.

## Conclusions

These data suggest that the peripheral level of MHPG (which is associated with noradrenaline levels in the brain) could be used as a biomarker for manic states in BDI. The MHPG level is likely to reflect the severity of the manic state in BDI, and noradrenaline may reflect the pathophysiology from manic to remission states. The MHPG level could be markedly different from individual to individual. Therefore, it is important that the MHPG level of an individual patient is known because it may change over time.
